# Differences in distribution and community structure of plant-parasitic nematodes in pecan orchards between two ecoregions of Georgia

**DOI:** 10.21307/jofnem-2021-075

**Published:** 2021-09-07

**Authors:** Ganpati B. Jagdale, Timothy B. Brenneman, Paul M. Severns, David Shapiro-Ilan

**Affiliations:** 1 Extension Nematology Lab, Department of Plant Pathology, University of Georgia, 2350 College Station Road, Athens, GA, 30602; 2 Department of Plant Pathology, University of Georgia, 2360 Rainwater Rd., Tifton, GA, 31793-5766; 3 Department of Plant Pathology, University of Georgia, 120 Carlton St, Athens, GA, 30602; 4 USDA, Agricultural Research Service, SE Fruit and Tree Nut Research Laboratory, 21 Dunbar Rd, Byron, GA, 31008

**Keywords:** Ecology, Host-parasite relationship, Multivariate analyses, Non-metric multi-dimensional analyses

## Abstract

In Georgia, pecans are commercially grown in the Piedmont and Coastal Plain ecoregions which are characterized by sandy-loam, sandy, and/or clay soils. If well-drained, these soils are suitable for pecan production, but the soil characteristics differ enough between ecoregions in which the plant-parasitic nematode (PPN) communities could differ substantially. We studied PPN communities in pecan orchards to evaluate the potential for ecoregion differences. In total, 11 genera (*Helicotylenchus*, *Hemicycliophora*, *Heterodera*, *Hoplolaimus*, *Meloidogyne*, *Mesocriconema*, *Pratylenchus*, *Paratylenchus*, *Paratrichodorus*, *Tylenchorhynchs*, *Xiphenema*) were recovered from pecan orchards in the Piedmont and Coastal Plain ecoregions. However, Non-Metric Multi-Dimensional Scaling ordination, Multi-Rank Permutation Procedure, and Indicator Species Analyses indicated that the pecan PPN communities strongly differed between ecoregions and that different genera were strongly associated with different ecoregions. For 9 of the 11 PPN genera, the maximum counts occurred in Coastal Plain locations, suggesting that the well-drained sandy soils of the Coastal Plain and comparatively ill-drained red clay soils of the Piedmont may be conducive and unfavorable for movement/reproduction of PPNs, respectively.

Nematodes are among the most ubiquitous, abundant, and biologically diversified groups of invertebrate soil organisms. Based on their feeding behavior, nematodes can be grouped as bacterivores, fungivores, predatory, carnivores, and herbivores (plant-parasites) (Pen-Mouratov et al., 2003; Yeates and Bongers, 1999; Yeates et al., 1993). All of these nematodes generally play key roles in ecological processes like nutrient recycling, decomposition of organic matter, and suppression of diseases (Briar et al., 2007; Ferris et al., 2004; Mulder et al., 2003; Neher, 2001, 2010; Yeates, 1999; Yeates and Bongers, 1999). Nematode species abundance and occurrence, and community composition are known to be influenced by both abiotic (physical and chemical properties of soil, temperature, and moisture) and biotic (e.g. host plant occurrence and abundance) factors (Bakonyi and Nagy, 2000; Freckman and Ettema, 1993; Kandji et al., 2001; Norton, 1989; Yeates, 1999). In agricultural systems, cultural practices like tillage, crop rotation, and addition of inputs can alter physical and chemical properties of soil that in turn can modify abundances and community structures of nematodes (Porazinska et al., 1999; Timper et al., 2012). For example, incorporation of organic soil amendments and fertilizers, such as compost, can change soil properties which favor the increase in bacterivores nematodes, which shifts the nematode community, but also promotes N-mineralization in the soil that in turn can increase the crop productivity (Neher, 1999).

For plant-parasitic nematodes (hereafter PPNs), they are obligately tied to the presence and relative abundance of host plants, but like other nematodes, their relative abundances may be influenced by soil properties such as the relative amounts of sand, slit, and clay (Van Gundy, 1985). For example, high populations densities of *Meloidogyne* spp. are commonly found in sandy soils over those soils with a large percentage of clay (Prot and Gundy, 1981), *Xiphenema americanum* densities were greater in silty clay-loam soils than silt-loam soils (Schmitt and Norton, 1972), and high population densities of *Pratylenchus* and *Criconemella* are typically found in fine silt and sandy soils, respectively (Wallace et al., 1993).

Over their North America distribution, pecan trees can be harmed with diminished yields from PPNs, especially three species of root-knot nematodes (RKN) *Meloidogyne incognita* (Hendrix and Powell, 1968), *M. arenaria* (Carithers, 1978) and *M. partityla* (Kleynhans, 1986), and ring nematodes (*Mesocriconema xenoplax*) (Nyczepir and Wood, 2008). In Georgia, pecans are primarily grown in two ecoregions, the Piedmont and Coastal Plains with estimated yields across the state totaling over 45 million kg and over $401 million pecan farm gate value obtained from over 177,000 acres planted in 2017 (Anonymous, 2017, 2019). Over the last 11 years, the Nematode Diagnostic Laboratory, University of Georgia, Athens had received and processed only 14 soil samples from pecan growers. These samples contained seven PPN genera including *Meloidogyne*, *Mesocriconema*, stubby-root (*Paratrichodorus*), stunt (*Tylenchorhynchs*), spiral (*Helicotylenchus*), lance (*Hoplolaimus*), and dagger (*Xiphenema*) nematodes (Jagdale pers. obs.). Of these seven PPN genera, only *Meloidogyne* spp. and *Mesocriconema* are known to cause damage to pecans (Nyczepir and Wood, 2008). Interpretation of PPN-pecan associations is further complicated by soil differences in the two biogeographic regions in which pecans are grown. The Piedmont ecoregion is located in the central part of Georgia between the Appalachian mountain foothills of North Georgia and the fall line, and is dominated by sandy loam soil with 43–85% sand, 0–50% silt, and 0–25% clay (Markewich et al., 1990; Pederson and Lathem, 1999), and red clay soil containing 40–50% sand, 10–15% silt, and 35% clay (Dr. Lessl personal communication, UGA Soil Test Lab). The Coastal Plain is divided into upper and lower subregions that occupy the southern part of Georgia between the fall line and the coast of Atlantic Ocean, and it is primarily sandy soil with 85–100% sand, 0–15% silt, and 0–10% clay, and clay soil with > 40% clay, < 45% sand, and < 40% silt (Markewich et al., 1990; Pederson and Lathem, 1999). Sandy-loam soils with a permeable clay subsoil is considered ideal for high pecan yields due to their good drainage and water holding capacities, yet the soil composition differences between ecoregions could generate PPN community differences that may require different control practices.

Based on the relative abundance of these PPN genera from the limited number of soil samples processed in the University of Georgia Diagnostic lab, and the potential that the sandy soils of the Coastal Plains may be more favorable for certain PPN genera, we used multivariate statistical tests, which are frequently used in community ecology studies, to determine whether the pecan nematode assemblages differed between the Piedmont and Coastal Plains ecoregions and which nematodes statistically contributed to any between ecoregion differences. Our findings could have implications for planning and implementing appropriate nematode control strategies in Georgia pecans.

## Materials and methods

### Selection of pecan orchards

Working in conjunction with Cooperative Extension agents and pecan growers, PPNs were sampled in 6 and 22 commercial pecan orchards located in four and 21 Georgia counties from Piedmont and Coastal Plain regions, respectively (Tables 1–3). Actively managed pecan orchards were selected from each county with possible symptoms (stunted growth and loss of vigor) of PPN infestations. In each orchard, 7–14 individual pecan trees were arbitrarily selected for soil sampling.

**Table 1.** Plant-parasitic nematodes found in commercial pecan orchards in Georgia in 2017, 2018, and 2019.

County	Nematode genera and ecoregions
*Piedmont region*	
Clarke	*Meloidogyne, Paratrichodorus, Mesocriconema, Helichotylenchus, Xiphinema, Paratylenchus, Hemicycliophora*, *Heterodera*
Rockdale	*Meloidogyne, Paratrichodorus, Mesocriconema, Helichotylenchus, Xiphinema*
Spalding	*Meloidogyne, Mesocriconema, Tylenchorhynchus, Helichotylenchus, Xiphinema*
Walton	*Meloidogyne, Hoplolaimus, Pratylenchus, Paratrichodorus, Mesocriconema, Tylenchorhynchus, Helichotylenchus, Hemicycliophora, Xiphenema*
*Coastal Plain region*	
Appling	*Meloidogyne, Hoplolaimus, Paratrichodorus, Mesocriconema, Helichotylenchus*
Berrien	*Meloidogyne, Paratrichodorus, Mesocriconema, Helichotylenchus*
Bibb	*Meloidogyne, Pratylenchus*, *Paratrichodorus, Mesocriconema*, *Helichotylenchus*, *Xiphinema*
Colquitt	*Meloidogyne, Paratrichodorus, Mesocriconema*, *Tylenchorynchus*, *Helichotylenchus*, *Xiphinema*
Cook	*Paratrichodorus, Mesocriconema*, *Xiphenema*
Crisp	*Meloidogyne, Paratrichodorus, Mesocriconema*, *Helichotylenchus*, *Xiphinema* and *Heterodera*
Decatur	*Meloidogyne, Paratrichodorus, Mesocriconema*, *Helichotylenchus*, *Xiphinema*
Dougherty	*Meloidogyne, Hoplolaimus, Paratrichodorus, Mesocriconema, Helichotylenchus, Xiphinema, Paratylenchus*
Grady	*Paratrichodorus, Mesocriconema, Helichotylenchus, Xiphinema*
Houston	*Meloidogyne, Hoplolaimus, Pratylenchus, Paratrichodorus, Mesocriconema, Helichotylenchus, Xiphinema*
Irwin	*Meloidogyne, Paratrichodorus* and *Mesocriconema*
Jefferson	*Meloidogyne, Paratrichodorus, Mesocriconema*, *Tylenchorhynchus, Helichotylenchus*, *Xiphinema*
Lee	*Meloidogyne*, *Mesocriconema*
Mitchell	*Meloidogyne, Hoplolaimus, Pratylenchus, Paratrichodorus, Mesocriconema, Helichotylenchus,Xiphinema, Paratylenchus*
Peach	*Meloidogyne, Hoplolaimus, Pratylenchus, Paratrichodorus, Mesocriconema, Tylenchorhynchus, Helichotylenchus, Xiphinema*
Taylor	*Meloidogyne, Paratrichodorus, Mesocriconema, Xiphinema*
Tift	*Meloidogyne, Paratrichodorus, Mesocriconema, Tylenchorhynchus, Paratylenchus*
Turner	*Meloidogyne, Paratrichodorus, Mesocriconema, Helichotylenchus, Xiphinema, Paratylenchus, Hemicycliophora*
Ware	*Meloidogyne, Hoplolaimus, Paratrichodorus, Mesocriconema, Helichotylenchus* and *Xiphinema*
Wilcox	*Meloidogyne, Paratrichodorus, Mesocriconema, Helichotylenchus,Xiphinema*
Worth	*Meloidogyne, Paratrichodorus, Mesocriconema, Xiphinema*

**Table 2.** Survey of frequency and abundance of five major nematode genera on pecans in Georgia during 2017–2019 by county in two pecan growing ecoregions, Piedmont (P) and Coastal Plain (CP).

		*Meloidogyne*			*Paratrichodorus*			*Mesocriconema*			*Helichotylenchus*			*Xiphinema*		
County	*N*	PF^a^	AB^b^	MD^c^	PF^a^	AB^b^	MD^c^	PF^a^	AB^b^	MD^c^	PF^a^	AB^b^	MD^c^	PF^a^	AB^b^	MD^c^
Clarke (P)	10	80	27	160	10	3	3	30	16	37	30	1	1	20	4	5
Rockdale (P)	10	10	7	7	20	2	2	80	3	6	100	20	69	70	1	1
Spalding (P)	10	30	24	50	–^d^	–	–	40	2	2	100	23	111	30	1	1
Walton (P)	30	33	21	124	23	4	10	100	8	31	87	27	97	27	2	3
Appling (CP)	10	90	13	23	80	3	14	100	10	33	10	1	1	–	–	–
Berrien (CP)	09	44	188	658	22	2	3	56	16	41	11	1	1	–	–	–
Bibb (CP)	10	20	36	58	100	7	13	100	24	97	100	206	637	50	2	5
Colquitt (CP)	07	71	41	83	100	4	9	100	44	89	71	13	25	14	2	2
Cook (CP)	10	–	–	–	50	2	3	100	10	71	–	–	–	10	5	5
Crisp (CP)	12	58	11	43	58	3	5	83	14	49	58	28	100	42	2	5
Decatur (CP)	10	70	27	78	20	1	1	90	5	12	10	1	1	60	2	5
Dougherty (CP)	10	50	5	11	50	3	5	100	24	45	20	2	2	20	1	1
Grady (CP)	10	–	–	–	20	1	1	90	18	46	60	3	4	40	6	14
Houston (CP)	14	43	14	34	14	2	2	93	54	293	14	16	30	36	3	9
Irwin (CP)	10	40	38	67	40	2	3	100	49	309	–	–	–	–	–	–
Jefferson (CP)	10	70	10	60	70	3	8	90	40	121	10	1	1	40	1	1
Lee (CP)	10	30	2	2	–	–	–	100	11	26	–	–	–	–	–	–
Mitchell (CP)	10	40	5	12	70	4	14	90	37	121	50	3	5	10	2	2
Peach (CP)	20	15	35	84	95	10	29	85	18	72	95	27	187	50	2	4
Taylor (CP)	10	40	1	1	20	3	4	90	6	27	–	–	–	20	3	3
Tift (CP)	10	70	11	36	60	3	3	100	20	49	–	–	–	–	–	–
Turner (CP)	10	30	39	104	10	1	1	100	20	62	40	2	6	90	3	5
Ware (CP)	10	20	3	4	10	1	1	100	11	31	10	1	1	20	2	3
Wilcox (CP)	10	70	22	119	30	4	6	90	16	36	10	13	13	10	1	1
Worth (CP)	10	80	18	64	10	1	1	100	28	60	–	–	–	40	2	4

**Notes:**^a^PF = percent frequency‒Total number of samples in which nematode genera was detected divided by the total number of samples collected in that county (*N*), multiplied by 100 to convert to a percentage. ^b^AB = Abundance**‒**Sum of nematode densities per 100 cm^3^ soil divided by the total number of samples in which the nematode genus was detected in the county on either survey date. ^c^MD = Maximum density of each genera detected per 100 cm^3^ soil. ^d^Dashes indicate the absence of specific nematode genera.

**Table 3.** Frequency of occurrence and abundance of plant-parasitic nematodes in commercial pecan orchards from two ecoregions of Georgia, 2017–2019.

	Coastal Plain region								Piedmont region							
					Mean percentages of 6 soil samples								Mean percentages of 4 soil samples			
Nematode genus	PF^a^	ABN^b^	STD	MD^c^	Sand	Silt	Clay	pH	PF^a^	ABN^b^	STD	MD^c^	Sand	Silt	Clay	pH
*Heterodera*	2	35	9.3	138	87.5	5.92	6.56	6.6	3	4	0.7	5	65.5	17.25	17.28	5.45
*Xiphinema*	28	3	1.6	14	87.5	5.92	6.56	6.6	33	2	1.0	5	65.5	17.25	17.28	5.45
*Hoplolaimus*	7	4	1.5	15	87.5	5.92	6.56	6.6	3	2	0.4	2	65.5	17.25	17.28	5.45
*Pratylenchus*	1	1	0.1	1	87.5	5.92	6.56	6.6	13	4	1.5	7	65.5	17.25	17.28	5.45
*Paratylenchus*	1	6	2.9	42	87.5	5.92	6.56	6.6	3	4	0.7	5	65.5	17.25	17.28	5.45
*Mesocriconema*	93	23	35.2	309	87.5	5.92	6.56	6.6	75	7	7.2	37	65.5	17.25	17.28	5.45
*Meloidogyne*	44	25	47.6	658	87.5	5.92	6.56	6.6	37	23	27.0	160	65.5	17.25	17.28	5.45
*Hemicycliophora*	1	1	0.1	1	87.5	5.92	6.56	6.6	15	3	1.3	6	65.5	17.25	17.28	5.45
*Helichotylenchus*	30	44	58.6	637	87.5	5.92	6.56	6.6	82	23	27.7	111	65.5	17.25	17.28	5.45
*Paratrichodorus*	46	5	4.2	29	87.5	5.92	6.56	6.6	17	3	1.6	10	65.5	17.25	17.28	5.45
*Tylenchorhynchus*	4	23	11.1	164	87.5	5.92	6.56	6.6	8	2	0.9	6	65.5	17.25	17.28	5.45

**Notes:**^a^PF (Percent frequency) = Percent of total samples with species present, *N* = 222 and 60 samples from Coastal Plain and Piedmont regions, respectively. ^b^ABN (Abundance) = Sum of nematode densities per 100 cm^3^ soil divided by the total number of samples in which the nematode genus was detected. ^c^MD (Maximum density) = Maximum count observed in 100 cm^3^ soil samples. Minimum was zero for all species. STD = standard deviation.

### Soil sampling

Each tree was then sampled by removing 10 random soil cores (15 cm deep x 2.5 cm diam) under canopy drip line around tree stem (Khanal et al., 2016). Soil cores from each tree were combined into one composite sample and 7–14 such composite samples were collected for each orchard. A total of 282 composite samples collected from all the selected pecan orchards and each composite sample was placed in a plastic bag and transported back to the Extension Nematology Laboratory, Athens, Georgia in coolers to assay nematode populations.

### Extraction, identification, and counting of nematodes

Plant-parasitic nematodes were collected from a 100 cm^3^ soil sub-sample taken from each of 282 composite samples using centrifugal sugar floatation technique (Jenkins, 1964). Nematodes from each sample were identified to genus level using diagnostic keys by Mai et al. (1996) and counted at 40X magnification using an inverted compound microscope. Then their frequency of occurrence, abundance, and maximum population densities were calculated using standard formulas (see Tables 2 and 3 captions).

### Soil properties

Soil pH and texture (proportions of sand, silt, and clay) analyses were conducted with four samples from two representative pecan orchards (Clarke and Walton counties – Piedmont ecoregion) and with six samples from four pecan orchards (Dougherty, Houston, Peach, and Tift counties – Coastal Plain ecoregion). A total of 10 soil samples were analyzed at the University of Georgia soil test laboratory in Athens, Georgia using standard methods (Gee and Bauder, 1986; Thomas, 1996).

### Statistical analysis of ecoregion PPN communities

We used a trio of multivariate analyses to evaluate the potential for ecoregion associated pecan PPN community differences. First, we used Non-Metric Multi-Dimensional Scaling (NMS or NMDS) (Kruskal, 1964; McCune and Grace, 2002), an ordination analysis that more accurately represents the structure within and between biological communities that PCA and PCoA (McCune et al., 2002), to visualize the patterns of PPN abundance and occurrence in soil samples collected from the Piedmont and Coastal Plain. NMS analysis was conducted using two data matrices, a primary and a secondary matrix. The primary matrix consisted of count data (relative abundance) from all 11 nematode from each pecan tree composite sample. Because nematode relative abundance varied by several orders of magnitude between genera and some genera were not present in a composite sample (a zero), we *ln* (*x* + 1) transformed the count data to conserve relative abundance (adding a 1 to all values enables us to *ln* transform all data while maintaining the zero in the data; *ln* 1 = 0, *ln* 0 is undefined and not appropriate for statistical analyses) and conform to multi-variate normality. Data from the primary, *ln x* + 1 transformed matrix, were converted to Gower’s distance values (Gower, 1971) for ordination. These values index the relative distance between the nematode counts for each pecan tree by simultaneously accounting for both PPN presence and relative abundance. NMS produces a scatterplot of how the pecan trees are related to each other given the Gower distance of each composite sample and calculates the centroid for each PPN genus in multivariate space. A secondary matrix was used to group the pecan tree PPN samples by ecoregion, either Piedmont or Coastal Plain. In ordination analyses, the more distant or closer the points are to each other in ordination space, the more dissimilar or similar the PPN communities in those samples are relative to each other, respectively. If there are differences in the PPN communities associated with ecoregion, we expect the NMS ordination to group pecan composite samples by ecoregion. NMS was accomplished through 500 real runs and 500 randomized runs in the program PC-ORD 7 (McCune and Mefford, 2016).

To test for statistically significant differences between the Piedmont and Coastal Plant pecan PPN communities, we retained the primary matrix (*ln x* + 1 transformed) and secondary matrix in the NMDS analysis and used them both for Multi-Rank Permutation Procedure (MRPP) (Mielke and Berry, 2001). MRPP is similar to an ANOVA but it is used for multivariate data. It evaluates group membership by comparing the distance values from each sample in the a priori selected groups (Piedmont and Coastal Plains ecoregions), calculates a test statistic for the between group differences (*T*, analogous to a student’s *t*), and indexes the effect size though a chance agreement within groups (A-statistic). A *p*-value for between groups statistical significance is obtained through the T-statistic (Mielke and Berry, 2001). MRPP was run from Gower’s Index values (Gower, 1971) in the program PC-ORD 7.

Because MRPP only tests for group membership, e.g. whether the pecan PPN communities differed between Piedmont and Coastal Plain ecoregions, we applied Indicator Species Analysis (ISA) (Dufrêne and Legendre, 1997) to identify which PPN genera, if any, were statistically associated with either ecoregion. Indicator values range from 0 to 100, with a 100 indicating a perfect indicator species (one that is mutually exclusive to a group and always occurs in the highest relative abundance within that group) and zero (a species that is not affiliated with any group either through occurrence or relative abundance) (see Severns and Sykes, 2020 for a description of the analysis). Probability values of ecoregion association were attained through 5,000 randomizations in PC-ORD 7.

## Results

### Patterns of pecan PPN diversity and distribution

The distribution of 11 PPN genera in six and 22 pecan orchards varied among the four and 21 counties that located in Piedmont and Coastal Plain ecoregions, respectively (Table 1). Also, the percent frequencies of occurrence, abundances, and maximum population density of five major PPNs including *Meloidogyne*, *Mesocriconema, Paratrichodoru, Helicotylenchus*, and *Xiphenema* that occurred on pecans differed between the nematodes and counties that are located in two different ecoregions of Georgia (Table 2). For example, only *Mesocriconema* was found in all locations in both ecoregions with its percent frequency of occurrences ranging from lowest (30%) to highest (100%). Of the four counties surveyed in the Piedmont region, PPN genera including *Meloidogyne*, *Xiphenema*, and *Helicotylenchus* found in all four counties with lowest (10%) and highest ( > 70%) of frequencies of occurrence but *Paratrichodorus* found in only three counties with only 10–23% of frequencies of occurrence (Table 2). Of the 21 counties surveyed in the Coastal Plain region, PPN genera including *Paratrichodorus*, *Meloidogyne*, *Xiphenema*, and *Helicotylenchus* found in 20, 19, 16, and 15 counties, respectively with lowest (10%) and highest (> 70%) of frequencies of occurrence (Table 2).

### Ecoregion soil properties and ecoregion associated differences in pecan PPNs

The distribution of 11 PPN genera that were associated with pecans in both geographic regions of Georgia varied between Coastal Plain and Piedmont ecoregions (Table 3). Also, the differences in frequencies of occurrences of PPNs and their communities are correlated with the properties of soil from both ecoregions (Table 3). For example, the percent frequencies of occurrence of ring (93%) and root-knot (44%) nematodes were comparatively higher in Coastal Plain, which is dominated with sandy soil containing average of 87.52% sand, 5.92% silt, and 6.56% clay with a pH of 6.6 (averages of six samples) than those in the Piedmont ecoregion (75 and 37%, respectively), which is dominated with sandy-clay-loam soil containing average 65.47% sand, 17.25% silt, and 17.28% clay and a mean pH of 5.5 (averages of four samples). In contrast, the percent frequencies of occurrence of dagger (33%) nematodes was higher in the Piedmont than in the Coastal Plain ecoregion (28%) (Table 3). The maximum population densities of nine nematodes were comparatively higher in Coastal Plain with sandy soil than those in the Piedmont ecoregion with sandy-clay-loam soil (Table 3).

**Figure 1:**
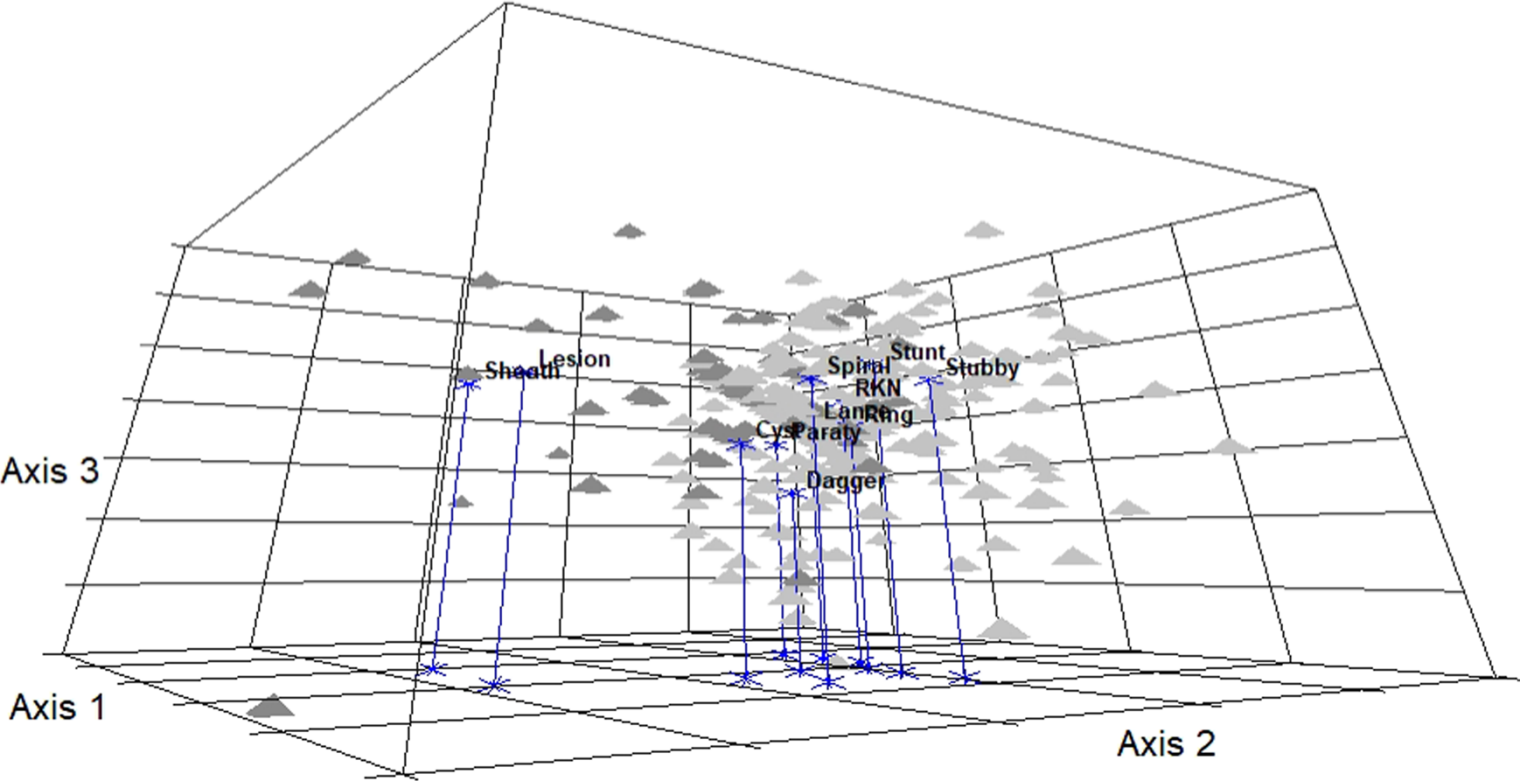
3-D NMS ordination of PPN soil samples from 282 Pecan trees in the Piedmont and Coastal Plain ecoregions of Georgia, USA (Final stress of the 3-D NMS solution = 13.36, proportion of variance explained by all three axes combined = 0.911). Dark gray pyramids (▲) represent pecan soil samples collected from the Piedmont ecoregion (*n* = 60) and light gray pyramids (▲) represent soil samples collected from the Coastal Plant ecoregion (*n* = 222).Blue asterisk with names in bold text represent the centroids of the 11 PPN genera (sheath, lesion, cyst, dagger, lance, ring, RKN (root-knot), spiral, stunt, stubby root, pin (*Praty*)) in the 3-D NMS solution. Piedmont ecoregion soil samples were distributed from the left side of the NMS ordination space to the middle (front to back). Coastal Plain ecoregion soil samples were distributed primarily from the middle to the right side of ordination space. This spatial division of soil samples taken from the Piedmont and Coastal Plains ecoregions strongly suggests that the pecan PPN communities differ between ecoregions.

NMDS ordination of the Georgia pecan PPN community yielded a three-dimensional solution that explained 91.9% of the variation in the soil sample data. The final stress of the 3-d solution was 13.36, a relatively stable NMDS ordination for ecological communities (McCune and Grace, 2002). Composite soil samples from the Piedmont ecoregion occurred primarily on one side of the ordination (left), while samples from the Coastal Plain occurred primarily on the opposite side (Fig. 1). In the Piedmont, the centroids (asterisks in Fig. 1) of lesion and sheath nematodes occurred in the center of the Piedmont samples (dark gray pyramids), suggesting these PPNs were proportionately dominant in the Piedmont. The centroids for stubby root, dagger, and stunt were positioned within the Coastal Plain samples (dark gray pyramids), while the centroids for ring, RKN (root-knot nematode), cyst, and spiral were not as obviously associated with either ecoregion (Fig. 1).

MRPP indicated that the pecan PPN communities statistically differed between the two ecoregions (MRPP results: *T* = 3.083, *A* = 0.043, observed delta value = 0.115, *p* < 0.000000001). Indicator Species Analysis (ISA) identified that ring and stubby-root nematodes were statistically associated with Coastal Plain samples and that spiral, sheath, and lesion nematodes were statistically associated with the Piedmont ecoregion samples (Table 4 ISA). The PPNs that the Indicator Species Analysis identified as being statistically associated with either the Piedmont or Coastal Plain ecoregions are likely those that contributed to the statistical differences of PPN communities between ecoregion indicated by MRPP.

**Table 4.** Indicator Species Analysis (see Severns and Sykes, 2020 for an analysis description) results showing which PPN nematodes were statistically associated (*) with pecan soil samples from the Piedmont (60 soil samples) or Coastal Plain (222 soil samples) ecoregions of Georgia, USA.

Nematode genus	Indicator value (0–100)	Ecoregion	*p* value (from 5,000 randomizations)
Ring, *Mesocriconema* spp.*	73.8	Coastal Plain	0.0002
Spiral, *Helichotylenchus* spp.*	48.1	Piedmont	0.0002
Stubby-root, *Paratrichodorus* spp.*	36.1	Coastal Plain	0.0028
Root-knot, *Meloidogyne* spp.	24.4	Coastal Plain	0.69
Dagger, *Xiphinema* spp.	15.3	Piedmont	0.85
Sheath, *Hemicycliophora* spp.*	14.8	Piedmont	0.0002
Lesion, *Pratylenchus* spp.*	13.0	Piedmont	0.0002
Lance, *Hoplolaimus* spp.	5.8	Coastal Plain	0.28
Pin, *Paratylenchus* spp.	3.6	Coastal Plain	0.64
Stunt, *Tylenchorhynchus* spp.	3.3	Coastal Plain	0.83
Cyst, *Heterodera* spp.	2.0	Coastal Plain	0.71

## Discussion

The first systematic survey of pecan PPNs in Georgia indicated that 11 genera (*Helicotylenchus*, *Hemicycliophora*, *Heterodera*, *Hoplolaimus*, *Meloidogyne*, *Mesocriconema*, *Pratylenchus*, *Paratylenchus*, *Paratrichodorus*, *Tylenchorhynchs*, and *Xiphenema*) occur throughout the Piedmont and Coastal Plain ecoregions. Within an ecoregion, the pecan PPN communities appeared similar in the NMDS ordination analysis, but the communities also appeared to differ between ecoregions in their composition as samples from the Piedmont grouped together while those from the Coastal Plain generally grouped together and separately from the Piedmont.

The MRPP analysis indicated that the pecan PPN communities had strong statistical differences between the ecoregions. While a relatively new statistical test of association, ISA is rarely used in plant disease studies but it has successfully identified known PPNs causing plant disease from field samples (Severns et al., 2020) and revealed multiple causal agents in an emerging plant disease (Rivedal et al., 2020; Severns and Sykes, 2020). Indicator Species Analysis in the present study clearly identified several PPN genera that had strong statistical associations with one of the two ecoregions. We also found that the maximum population densities of 9 out of 11 PPN genera were comparatively higher in the Coastal Plain than those occurring in the Piedmont. These ecoregion differences in the pecan PPN community and ecoregion-specific association with individual PPN genera may be due to soil texture characteristics that are known to influence PPN distribution and abundance (Norton and Hoffmann, 1974; Norton et al., 1971). The well-drained and coarse, sandy soils in the Coastal Plain (Markewich et al., 1990) and comparatively ill-drained in the Piedmont (Markewich et al., 1990) may be conducive and unfavorable for movement/reproduction/development of PPNs, respectively (Anonymous, 2018; Jones et al., 1969; Kim et al., 2017; Koenning et al., 1996; Pang et al., 2011; Sasser, 1954; Seshadari, 1964). Other soil factors (e.g., nutrients or organic matter) may also affect PPN populations (Noe and Barker, 1985; Norton et al., 1971), but those soil traits were not measured in our study. We recognize that there were fewer replicate sites for the Piedmont ecoregion compared with the Coastal Plain, yet the six replicate sites in the Piedmont are considered appropriate for valid analyses and conclusions. Nonetheless, future studies may expand upon our study and utilize more sites and replicates from each ecoregion.

Our results agree with the findings of previous researchers, who reported greater PPN population densities of nine genera (cyst, dagger, lance, pin, ring, root-knot, spiral, stubby-root, and stunt nematodes) in the rhizospheres of different crops in sandy compared to clay soils (Brodie, 1976; Dropkin, 1980; Koenning et al., 1996; Lewis and Smith, 1976; Martin et al., 1994; Olabiyi et al., 2009; Wallace et al., 1993). In contrast, the population density of root-lesion nematode, *Pratylenchus* spp. (Davis and MacGuidwin, 2005) appeared to be greater in sandy loam Piedmont soils than in the sandy soils of the Coastal Plain. These results are consistent with previous researchers who also reported high population densities of lesion nematodes infecting different crops in sandy-loam soils (Endo, 1959; Florini et al., 1987; Jordaan et al., 1989; Salem et al., 1994).

Although we found 11 PPN genera in our pecan study, only *M. partityla* and *M. xenoplax* have been previously reported to be associated with- and pathogenic to pecans in Georgia (Nyczepir and Woods, 2008; Nyczepir et al., 2002, 2004a, b) and South Africa (Kleynhans, 1986). In our study comparing the nematode communities between the two ecoregions where pecans are most frequently grown, we found an additional nine genera in the pecan rhizosphere for which we do not yet know their specific taxonomic rank nor the pathogenic potential to pecans. In Georgia, Pecan RKN, *M. partityla* is considered the most damaging PPN as it is associated with typical mouse-ear foliar symptoms, nickel deficiencies, stunted growth and dead branches in the upper canopy of pecans (Nyczepir et al., 2002, 2006). *Meloidogyne partityla* has been also reported to be associated with weakening of pecan trees in other states such as Arizona (Khanal et al., 2016), Florida (Brito et al., 2006), New Mexico (Thomas et al., 2001), Oklahoma (Brito et al., 2006) and Texas (Starr et al., 1996), and South Africa (Kleynhans, 1986). Previously, association of two other species of RKNs including *M. arenaria* (Carithers, 1978) and *M. incognita* (Hendrix and Powell, 1968) have been reported in Georgia pecans, but Nyczepir and Woods (2008) could not confirm their recent presence in pecan orchards in Georgia. Also, *M. javanica* has been reported to be pathogenic to some cultivars of pecans (Pinochet et al., 1993). Furthermore, the occurrence of different RKN species *M. arenaria*, *M. javanica*, and *M. incognita* have been reported in the rhizosphere of different stone fruits like nectarine, peach, plum, and prune grown in California (Chitambar et al., 2018). In the present study, it appears that RKNs (*Meloidogyne* spp.) are widespread in pecan orchards located in 23 different Georgia counties (4 and 19 counties from both Piedmont and Coastal Plain ecoregions, respectively). However, the RKN species is unknown in Georgia as we have only confirmed the presence of *M. partityla* in three counties (Jefferson, Houston, and Tift) located in Coastal Plain ecoregion of Georgia (Waliullah et al., 2020; Jagdale unpublished data). Nyczepir and Woods (2008) reported a wide distribution of *M. partityla* in Georgia, but they did not provide any specific distribution details. Considering the known differences among these species of root knot nematodes (host range in particular), it will be important to positively identify the species of RKNs present in Georgia pecan orchards. Such information is critical to understanding the life cycle of pecan RKNs and formulating the most effective control strategies.

In the present study, *Mesocriconema* spp. was the most abundant and frequently occurring nematode (overall 89% of samples from both ecoregions) with a maximum mean population density of 309 nematodes/100 cm^3^ soil. These findings are consistent with Nyczepir et al. (2004b), who also reported the association of high numbers of ring nematodes with stressed and stunted pecan trees in Georgia. Although the damage threshold level of ring nematodes on pecan is unknown, it has been reported that *M. xenoplax* occur in large numbers in the rhizosphere soils of other nut crops and stone fruits including almond apricot, cherry, plum, prune, peach, and walnut (Anonymous, 2020a, b; Chitambar et al., 2018; Ciancio and Grasso, 1998; Dong et al., 2007; Lownsbery et al., 1974, 1978; Nyczepir, 1989). Also, the well-known association of *M. xenoplax* with the disease complex in peaches called peach tree short life (PTSL) (Nyczepir, 1989; Nyczepir et al., 1983) suggests that it may substantially impact Georgia pecan production as both of these crops are often planted in close proximity or inter-planted in Georgia (Cottrell et al., 2010; Smith et al., 1989).

Next to RKNs, spiral nematodes (*Helicotylenchus* spp.) were present in 41% soil samples with the highest population density of 637 nematodes/100 cm^3^ soil collected from the rhizosphere of pecans. Associations of five different species of spiral nematodes (*H. dihystera*, *H. digonicus*, *H. pseudorobustus*, *H. paragiris*, and *H. microlobus*) with different nut trees (almond, apricot, and walnut) and stone fruits (cherry, peach, plum, and nectarine) have been also reported in California and Greece (Chitambar et al., 2018; Tzortzakakis et al., 2018) but there is virtually no information available on the association of specific species of spiral nematodes with pecans. In general, different species of spiral nematodes are found in the rhizosphere of different field and fruit crops, ornamental plants, turfgrasses, weeds, and nut crops but they are not considered economically important pests on many host crops, excepting *H. multicinctus*, *H. paaxilli*, and *H. microlobus*, which are considered serious pests of banana (McSorley and Parrado, 1983) and turfgrasses (Jagdale et al., 2020; Pang et al., 2011, 2012). Since spiral nematodes are known to cause discolored lesions in the root cortexon which they feed (Jagdale et al., 2020; Maggenti, 1981), they may pose pathogenic potential to pecans but this requires confirmation.

In the rhizosphere of Georgia pecans, stubby-root and dagger nematodes were present in 39 and 29% soil samples with the highest population densities of 29 and 14 nematodes/100 cm^3^ soil, respectively. Both of these nematodes are considered economically important because they may vector various types of plant virus diseases. According to Brown et al. (1995), nepoviruses are transmitted by species in the genera *Xiphinema* whereas tobraviruses are transmitted by species of *Paratrichodoru*s. However, there are no reports of viral diseases vectored by these nematodes in pecans in the USA or elsewhere in the world, suggesting that monitoring and focused investigation is needed to confirm their potential in transmitting viruses to pecans.

Although both stunt (*Tylenchorhynchus* spp.) and cyst (*Heterodera* spp.) nematodes can cause damage to many field crops, there are no reports available on the occurrence of these nematodes on pecans. In the present study, the intermediate mean population densities of 164 and 138 nematodes/100 cm^3^ soil were recorded for stunt and cyst nematodes in pecans, respectively. Of these two nematodes, stunt nematodes are known to be associated with stone fruits and nuts grown in California (Chitambar et al., 2018) but nothing is known regarding the association of cyst nematodes either on nut crops or stone fruits (Lilley et al., 2005). Other nematodes including lance, lesion, pin, and sheath were encountered at a low frequency and population densities in the pecan rhizosphere and are not strong candidates as agents that could lower pecan yields.

## Conclusions

To the best of our knowledge, this is the first study that revealed associations between soil properties and PPN community structures in pecan orchards located in Piedmont and Coastal Plain ecoregions of Georgia. We demonstrated that 11 PPN genera were common to both ecoregions but their communities strongly differed in composition between the ecoregions. These ecoregion-associated differences appear to be related to soil texture and drainage. Further studies are needed to discover the influence of chemical properties of soil on the abundance and diversity of PPN communities of pecans and other crops in the region that in turn may help in planning IPM programs for overall pecan husbandry in Georgia.
